# Complete genome analysis of hepatitis B virus in Qinghai-Tibet plateau: the geographical distribution, genetic diversity, and co-existence of HBsAg and anti-HBs antibodies

**DOI:** 10.1186/s12985-020-01350-w

**Published:** 2020-06-12

**Authors:** He Liu, Liping Shen, Shuang Zhang, Feng Wang, Guomin Zhang, Zundong Yin, Feng Qiu, Xiaofeng Liang, Fuzhen Wang, Shengli Bi

**Affiliations:** 1 NHC Key Laboratory of Medical Virology and Viral Diseases, National Institute for Viral Disease Control and Prevention, Chinese Center for Disease Control and Prevention, Changbai Rd 155#, Changping District, Beijing, China; 2 Department of Infectious Disease, Tianjin Center for Disease Control and Prevention, Tianjin, People’s Republic of China; 3 National Immunization Program, Chinese Center for Disease Control and Prevention, Beijing, People’s Republic of China; 4 Chinese Center for Disease Control and Prevention, Beijing, People’s Republic of China

**Keywords:** Hepatitis B virus, Mutation/mutation rate, Recombination, Hepatitis B surface antigen, Antibody to HBsAg

## Abstract

**Background:**

The genetic variation and origin of Hepatitis B Virus (HBV) in Qinghai-Tibet Plateau were poorly studied. The coexistence of HBsAg and anti-HBs has been described as a puzzle and has never been reported in the indigenous population or in recombinant HBV sequences. This study aimed to report geographical distribution, genetic variability and seroepidemiology of HBV in southwest China.

**Methods:**

During 2014–2017, 1263 HBsAg positive serum were identified and 183 complete genome sequences were obtained. Serum samples were collected from community-based populations by a multistage random sampling method. Polymerase chain reaction (PCR) was used to amplify the HBV complete genome sequences. Then recombination, genetic variability, and serological analysis were performed.

**Results:**

(1) Of the 1263 HBsAg positive serum samples, there were significant differences between the distribution of seromarkers in Tibet and Qinghai. (2) Of 183 complete genome sequences, there were 130 HBV/CD1 (71.0%), 49 HBV/CD2 (26.8%) and four HBV/C2 isolates (2.2%). Serotype ayw2 (96.1%) was the main serological subtype. (3) Several nucleotide mutations were dramatically different in CD1 and CD2 sequences. Clinical prognosis-related genetic variations such as nucleotide mutation T1762/A1764 (27.93%), A2189C (12.85%), G1613A (8.94%), T1753C (8.38%), T53C (4.47%) T3098C (1.68%) and PreS deletion (2.23%) were detected in CD recombinants. (4) From the inner land of China to the northeast boundary of India, different geographical distributions between CD1 and CD2 were identified. (5) Twenty-seven (2.14%) HBsAg/HBsAb coexistence serum samples were identified. S protein amino acid mutation and PreS deletion were with significant differences between HBsAg/HBsAb coexistence group and control group.

**Conclusions:**

HBV/CD may have a mixed China and South Asia origin. Based on genetic variations, the clinical prognosis of CD recombinant seems more temperate than genotype C strains in China. The HBsAg/HBsAb coexistence is a result of both PreS deletion and aa variation in S protein. Several unique mutations were frequently detected in HBV/CD isolates, which could potentially influence the clinical prognosis.

## Background

Hepatitis B Virus (HBV) is considered to be a major global health problem with more than 250 million chronic HBV (CHB) carriers and more than one million HBV-associated human deaths per year [1]. In the nationwide investigation in 2006, Hepatitis B surface antigen (HBsAg) was identified in 7.2% of the whole population in China [2]. In Qinghai-Tibet Plateau, due to high altitude environment, religion issue, delayed vaccine inoculation or other unknown reasons, the HBV prevalence is over 10% according to our recent study (unpublished), which is much higher than in other areas of China. These carriers of HBV are at increased risk of developing liver cirrhosis (LC) and hepatocellular carcinoma (HCC) [3].

Qinghai-Tibet Plateau covers more than 2.5 million square kilometers, which is the second- largest plateau in the world and connecting south and northeast Asian [4]. In this area, a special recombinant of HBV genotype C and genotype D (HBV/CD) was reported [4]. However, the nature and origin of the recombinant are still poorly studied. At the same time, the coexistence of HBsAg and anti-HBs (HBsAb) in CHB patients has been occasionally reported [5, 6]. Due to the low incidence of HBsAg/ HBsAb coexistence in HBV carriers, the sample sizes in many previous studies [5, 7] were small (less than 20) and lack of adequate control subjects [8]. The mechanism underlying the emergence of anti-HBs in CHB patients remains unclear and related information has not been reported in the indigenous population or in recombinant HBV sequences.

To gain deeper insights into HBV genomic diversity of the special recombinants, 1263 HBsAg positive serum samples were obtained from eight areas of Qinghai-Tibet Plateau and 183 complete genome sequences of HBV isolates were under further analysis. Twenty-seven HBsAg/HBsAb coexistence serum samples were identified and studied with genome variation.

## Methods

### Procedures to detect point mutations and recombination of HBV by PCR and direct sequencing analysis

Sera samples from Qinghai-Tibet plateau → HBV infection serological markers detection→ DNA purification → HBV complete genome amplification → PCR-DNA purification → Automatic sequencing → Sequences assembling → Comparing with reference sequences.

### Subject sample collection

During 2014–2017, the subjects were collected from community-based populations from eight regions of Qinghai-Tibet Plateau based on the population density and covered most of the indigenous habitations, including Hainan, Lhasa, Shannan, Nyingchi, Ali, Nakqu, Chamdo and Rikaze. In this study, the multistage random sampling method was used to ensure the sample representativeness of the whole area. Firstly, two or three counties were selected at random from eight areas, respectively. Secondly, two villages were selected from every county. Thirdly, populations of 18–59 years old were selected from every village. Basic information was recorded on the questionnaire prepared beforehand, including name, gender, birth date, address, and medical information, and 5 mL of venous blood was taken from each participant. HBV infection markers including hepatitis B surface antigen (HbsAg), anti-hepatitis B surface antibody (HBsAb), anti-hepatitis B core antibody (HBcAb), hepatitis B e antigen (HBeAg) and anti-hepatitis B e antibody (HBeAb) were detected by chemiluminescent assays (AXSYM; Abbott Laboratories, North Chicago, IL, USA).

### HBV DNA extraction, whole-genome amplification, and sequencing

HBV DNA was extracted from 200 μL serum using QIAamp DNA Blood Mini Kit (Qiagen, Hilden, Germany). Full-length HBV DNA (about 3.2 kb) was amplified by Nested PCR finally performed in seven fragments. Based on the previously reported methods [9] used in our preliminary study [10], the primers and thermal profile were optimized in this study to adapt to HBV/CD isolates and identify more complete genomes. The basal core promoter (BCP) region was generated by two rounds. First round was conducted using the primer combination of BcpF1 and BcpR1 in a 25 μL reaction volume containing 5 μL extracted DNA and 12.5 μL premix Taq polymerase. The whole- genome (without BCP region) consisting of six fragments was also generated by two rounds of PCR. First round was conducted using the primer combination of HBV1799FLong and HBV1801RLong in a 50 μL reaction volume containing 15 μL extracted DNA and 25 μL premix Taq polymerase. All the primers and thermal profiles were listed in Table 1.

**Table 1:** List of primers used to amplify the different regions of the HBV genome and their respective thermal profile

HBV region	Round	Primer Name (Position)	Primer Sequence (5′-3′)	Thermal Profile
BCP/Precore region	First	BcpF1(1254–1273)	TCCTCTGCCGATCCATACTG	80 °C(3 min); 95 °C (40 s), 63 °C (1 min), 71 °C (2.5 min)/40 cycle; 72 °C(7 min)
		BcpR1(2416–2439)	TTCCCGAGATTGAGATCTTCTGCG	
	Second	BcpF2(1606–1625)	GCATGGAGACCACCGTGAAC	*95 °C(5 min); 95 °C (40 s), 50 °C (30 s), 72 °C (1 min)/35 cycle; 72 °C(7 min)*
		BcpR2(1955–1974)	GGAAAGAAGTCAGAAGGCAA	
Complete genome (without BCP)	First	HBV1799FLong(1799–1826)	CTGCGCACCAGCACCATGCAACTTTTTC	80 °C (3 min); 95 °C (40 s), 58 °C (1.5 min), 68 °C (4 min)/45 cycle; 72 °C(7 min)
		HBV1801RLong(1774–1801)	CAGACCAATTTATGCCTACAGCCTCCTA	
	Second			
	1	HBV1847FS (1847–1867)	TGTTCATGTCCCACTGTTCAA	95 °C(5 min); 95 °C (40 s), 63 °C (30 s), 72 °C (1 min)/35 cycle; 72 °C(7 min)
		HBV2394RS (2394–2408)	GGCGAGGGAGTTCTT	
	2	HBV2298FS (2298–2315)	GACCACCAAATGCCCCTAT	
		HBV2933RS (2933–2954)	TCGGGAAAGAATCCCAGAGGAT	
	3	HBV2821FS (2821–2840)	GGTCACCATATTCTTGGGAAC	
		HBV272RS (272–291)	TGAGAGAAGTCCACCACGAGT	
	4	HBV179FS (179–197)	CTAGGACCCCTGCTCGTGTT	
		HBV704RS (704–725)	CGAACCACTGAACAAATGGCACT	
	5	HBV0599FS(599–619)	GTATTCCCATCCCATCATCCTG	
		HBV1286RS (1286–1305)	GCTAGGAGTTCCGCAGTATGG	
	6	HBV1175FS (1175–1190)	GCCAAGTGTTTGCTGA	
		HBV1788RS(1788–1802)	GCCTACAGCCTCCTA	
		SP6	CGATTTAGGTGACACTATAGAAGAGAGGCT	
		T7	CAGTAATACGACTCACTATAGGGAGAAGGCT	

*Numbers within primer names represent the primer positions. An F after the primer position stands for sense primers while R stands for anti-sense primers

**SP6 and T7 are tag sequences attached at the 5′ end of PCR primers used in this study,except four Bcp primers. SP6 and T7 primers were used to sequence PCR fragments

After purification of the PCR products with a QIA Gel Extraction Kit (Qiagen, Valencia, CA), the sequences were determined using the Sanger dideoxy terminator sequencing method with DNA sequencer ABI 3700 (PE Applied Biosystem). Then the sequences were assembled using SeqMan II software (DNAStar Inc.), and the correct nucleotide position of the complete HBV sequence was revised through alignment with the reference sequence in the GenBank database.

The whole-genome nucleotide sequences reported in this article have been deposited in the National Center for Biotechnology Information GenBank database with accession numbers MN683570- MN683729, MN657315- MN657318, KX660674- KX660690.

### Recombination and serological analysis

Genotypes and recombination in genome sequences of HBV were investigated using SimPlot v3.5.1 software and JPHMM (jumping profile Hidden Markov Model) method [11]. MEGA 7.0 software was used to calculate genetic distance and pairwise distance comparisons. The HBsAg serotypes were deduced from the sequences of the S gene region by the identification of amino acids at positions 122 (Lys-Arg for d-y determinants), 160 (Lys-Arg for w-r determinants), 127 (Pro-Thr-Leu/Ile for w2-w3-w4), and in the case of Arg122 Pro127 Lys160, also at positions 159 (Ala-not Ala for ayw1-ayw2 and ayw4) and 140 (not Ser-Ser for ayw2-ayw4) [12].

### Mutation analysis

Generally, the mutation definition and analysis were performed as previously described [13]. The whole genome, including S gene, PreC/C gene, reverse transcriptase (RT), X gene and basal core promoter (BCP) were under nucleotide mutation analysis. Base on the structure of HBV/CD recombinants, genotype C and genotype D reference sequences were used as parental sequences for mutation detection [14, 15]. Nucleotide sequences of datasets in this study and reference HBV sequences were analyzed using the MEGA 7.0 software and Mutation Reporter Tool [16].

The nucleotide PreS/HBsAg sequences obtained were translated into amino acid sequences, aligned and compared with reference sequences. Amino acid variability was defined as the frequencies of residue substitutions at each position [17]. For analysis, HBsAg was divided into subregions corresponding to structural and/or functional domains: the N- terminal region (aa 1 to 99), the major hydrophilic region (MHR, aa 100 to 169) and the C- terminal region (aa 170 to 226). The “a” determinant in MHR (aa 124 to 147), the first loop of “a” determinant (aa 124 to 137) and second loop of “a” determinant (aa 139 to 147) were also analyzed respectively.

### Statistical analysis

Statistical analyses were performed using SPSS 22.0 (IBM Corp., Armonk, NY, USA). Chi-squared test and two-tailed Student’s *t*-test were used for analysis, as appropriate. A *P*-Value of ≤0.05 was considered as statistically significant.

### Ethical approval

The study protocol conformed to the ethical guidelines of the 1975 Declaration of Helsinki and was approved by the Ethics Committee of the Chinese Center for Disease Control and Prevention. The purpose of the study and the right to information were explained to the participants by research staff. Written informed consent was obtained from each participant before the interview and venous blood collection.

## Results

### Patient characteristics

During 2014–2017, 1263 HBsAg positive serum samples were identified from the community population of Qinghai-Tibet plateau. There were no differences in age (29.29 ± 17.93 vs 30.56 ± 18.98, *P* = 0.211) and gender (449/404 vs 201/210, *P* = 0.206) distribution between study population in Tibet and Qinghai. Of all the 1263 positive serum samples, twenty-seven serum samples (2.14%) were HBsAg/HBsAb coexistence. There were significant differences between the distribution of seromarkers in Tibet and Qinghai. Details of serological information are shown in Supplement Table 1.

One hundred and eighty-three samples were selected from the 1263 HBsAg-positive serum samples and then full-length sequences were amplified for analysis of the HBV genome. A total of 87 samples were HBeAg-negative, and 96 samples were HBeAg-positive. Most isolates had a genome size of 3215 nt, except eight sequences with evidence of deletion. Basic information of HBV/CD complete genome sequences was listed in Table 2.

**Table 2:** Basic information of HBV CD1 and CD2 isolates in Qinghai-Tibet Plateau

Character	CD1(130 isolates)	CD2(49 isolates)	*P* value
Gender (Sex, female/male)	63/67	21/28	0.503
HBeAg (positive/negative)	69/61	25/24	0.806
Age (median years ± SD)	27.51 ± 17.78	36.63 ± 17.71	0.006
DNA level (copies/mL ± SD)	6.79 ± 1.2	6.96 ± 1.5	0.089

### Recombination and serological results

**Fig. 1:**
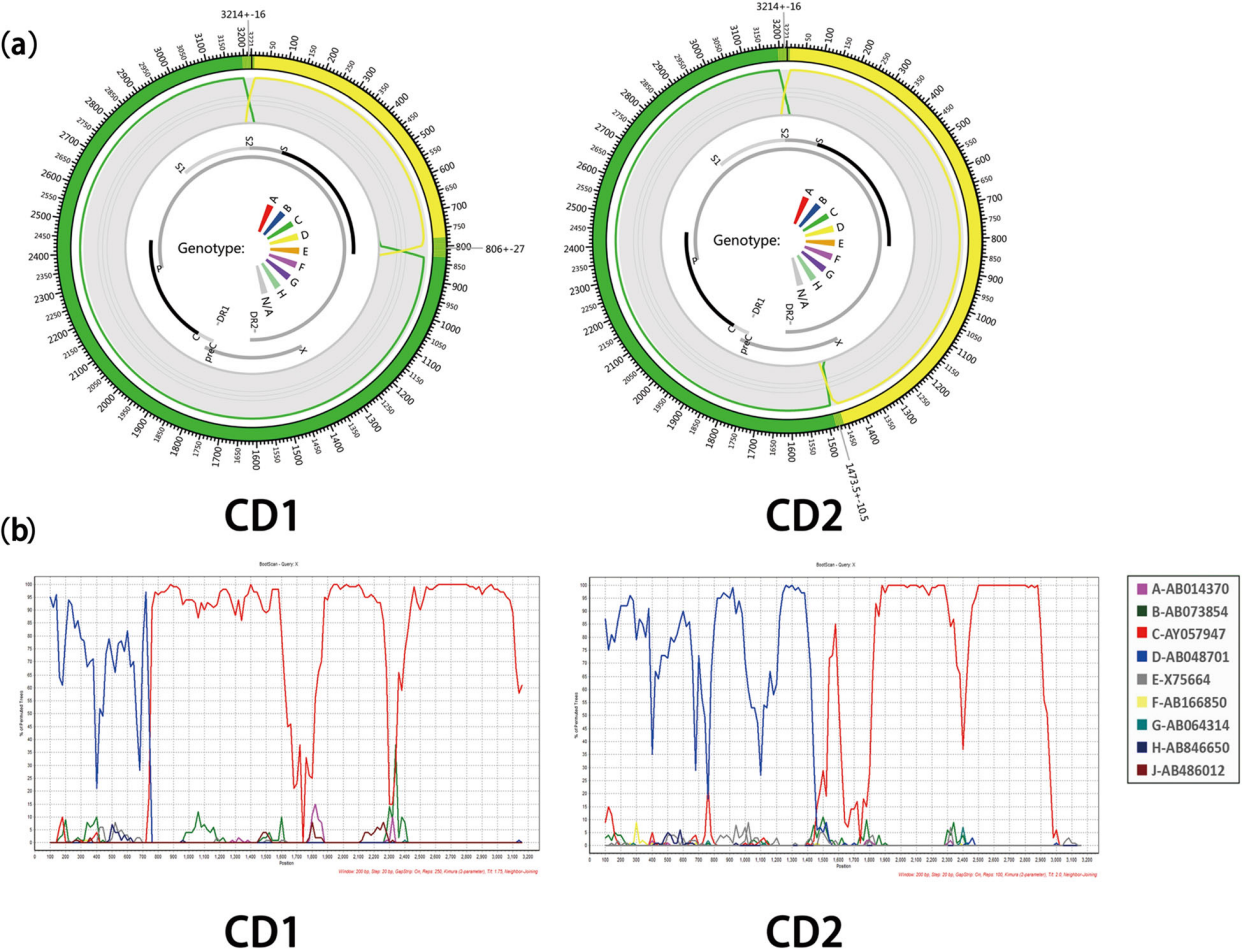
Genome recombination analyses in two types of HBV/CD isolates (XZ4-CD1, XZ109-CD2) using two approaches. **a** Schematic diagram of jpHMM results showing the circular HBV genome with alternate genotype C and genotype D regions. **b** Graphical representations of SimPlot and Bootscaning analysis demonstrate the recombination of genotypes C and D

There were two types of HBV/CD recombinants detected in this study. HBV/CD1 was with around nt10–800 from genotype D integrated into genotype C to form the recombinant viral strain; HBV/CD2 was with a region around nt10–1500 from genotype D integrated into genotype C to form the recombinant viral strain. Graphical display of JPHMM and Simplot recombination analysis results were shown in Fig. 1. Of all the 183 HBV complete sequences, there were 130(71.0%) HBV/CD1 isolates, 49 (26.8%) HBV/CD2 isolates and four (2.2%) HBV/C2 isolates. As the results of evolutionary divergence over sequences showed, the genotype D fragment of HBV/CD is closest to HBV subgenotype D4, while the genotype C fragment of HBV/CD is closest to the subgenotype C2 (Supplement Table 2 and 3).

The main serological subtype of HBV/CD is ayw2 (172/179, 96.1%). Of the 130 HBV/CD1 isolates, 126 (96.9%) were predicted to belong to serotype ayw2, two (1.5%) to ayr, one (0.8%) to ayw1 and one (0.8%) to ayw4. Of the 49 HBV/CD2 isolates, 46 (93.9%) belonged to ayw2 and three (6.1%) to adw2. All four HBV/C2 isolates belonged to adr serotype.

### Geographical distribution of HBV subgenotypes in the plateau

**Fig. 2:**
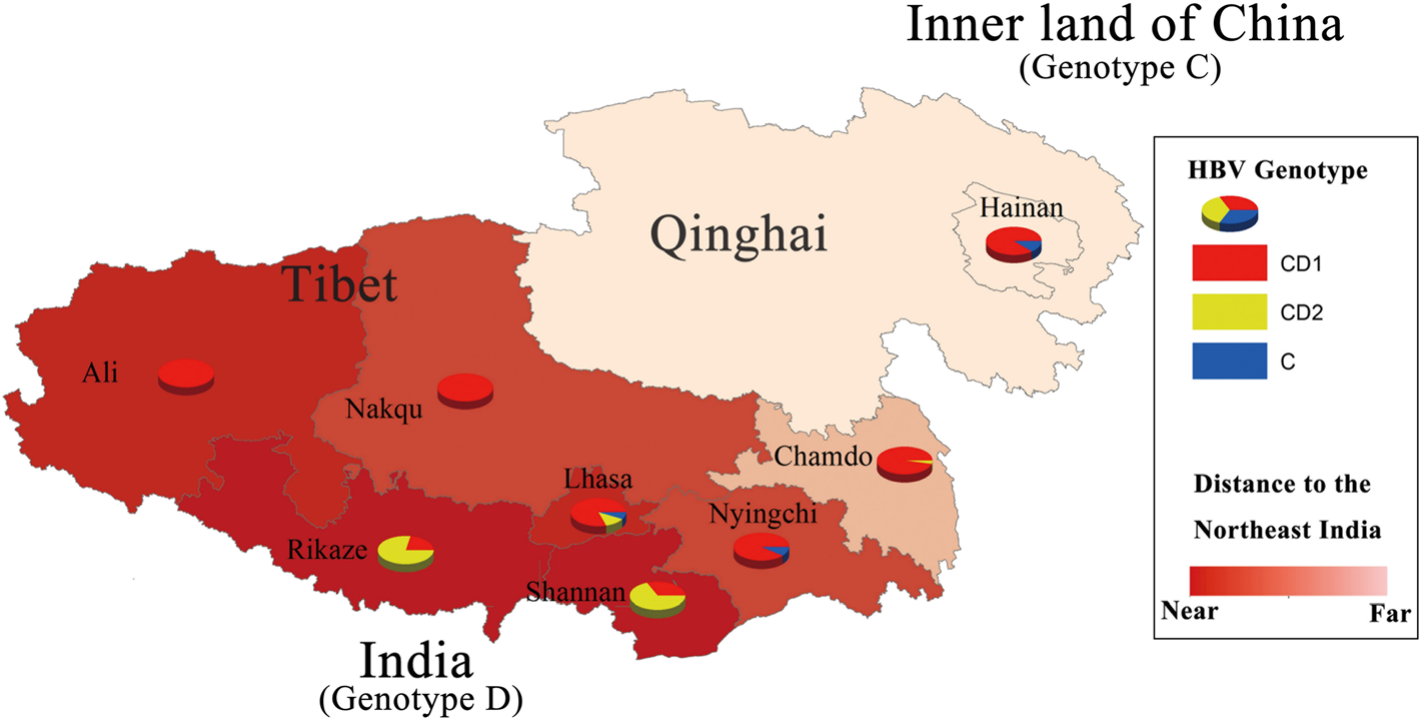
Distribution of HBV genotypes in eight study regions of Qinghai-Tibet Plateau. The proportion of the HBV recombinant CD1, CD2 and genotype C in each region is shown. The relative distance to the northeast of Indian is shown by the color of region in the map

HBV/CD1 recombinant sequences were predominantly identified in all the geographic regions analyzed in this study. However, most of the HBV/CD2 isolates (93.9%, 46/49) were identified in two regions (Shannan & Rikaze), which was significantly more than the other six regions in the plateau (*P* = 0). Four genotype C isolates were all identified in the eastern part of the plateau. The distribution of HBV subgenotypes in the plateau is shown in Fig. 2 and Supplement Table 4.

### Nucleotide mutations

**Fig. 3:**
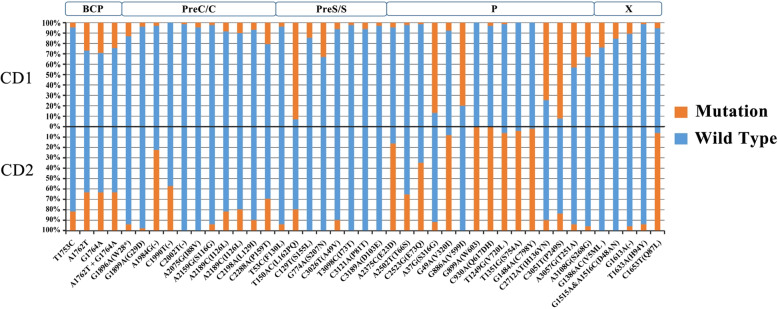
Distribution of wild type and nucleotide mutations (amino acid substitutions) in HBV/CD1 and HBV/CD2 genome. Each bar represents the percentage of isolates with mutated nucleotide (amino acid residues) in CD1 and CD2 recombinants

Four genotype C isolates in this study were not enough for statistical analysis, so only the HBV/CD recombinants (130 CD1 and 49 CD2) were under mutation analysis. In the level of nucleotide, the double mutations A1762T/G1764A in the BCP region of HBV (nt1742–1849) were frequently observed in HBV/CD sequences (50/179, 27.9%). In addition, T1753C, another mutation in BCP, was found at a frequency of 4.6% in CD1 and 18.4% in CD2 (*P* = 0.003). Compare to genotype C and genotype D reference sequences, there were different mutation patterns respectively detected in CD1 and CD2 isolates. Nucleotide mutations, including A1984G, T150AC, A2735C, C2523G, A37G, C2712AT, and C1653T were dramatically different in CD1 and CD2, though these mutations were in the same recombination regions (genotype C or genotype D fragment). Many nucleotide mutations cause amino acid change which could potentially change the clinical prognosis of the recombinants. Other mutations, including stop codon mutation G1896A, were identically detected in CD1 and CD2 isolates. Details of main nucleotide mutations and corresponding amino acid changes identified in the complete genome sequences of HBV/CD1 and HBV/CD2 were shown in Table 3 and Fig. 3.

**Table 3:** Main nucleotide and amino acid mutations of HBV CD1 and CD2 isolates in Qinghai-Tibet Plateau

Position	Nucleotide mutation	Amino acid mutation	CD1(130 isolates)		CD2(49 isolates)		Total (179 isolates)		*P* value
BCP	T1753C		6	4.62%	9	18.37%	15	8.38%	0.003
nt1742–1849	A1762T		35	26.92%	18	36.73%	53	29.61%	0.2
	G1764A		38	29.23%	18	36.73%	56	31.28%	0.334
	A1762T + G1764A		32	24.62%	18	36.73%	50	27.93%	0.107
PreC/C region	G1896A	W28*	17	13.08%	3	6.12%	20	11.17%	0.188
nt1814–2452	G1899A	G29D	5	3.85%	1	2.04%	6	3.35%	0.55
	A1984G		4	3.08%	38	77.55%	42	23.46%	< 0.001
	C1990T		0	0	21	42.86%	21	11.73%	< 0.001
	C2002T		2	1.54%	0	0	2	1.12%	0.383
	A2075G	I88V	6	4.62%	0	0	6	3.35%	0.126
	A2159G	S116G	3	2.31%	3	6.12%	6	3.35%	0.206
	A2189C	I126L	13	10%	10	20.41%	23	12.85%	0.064
	C2198A	L129I	9	6.92%	5	10.20%	14	7.82%	0.466
	C2288A	P159T	27	20.77%	15	30.61%	42	23.46%	0.166
PreS/S gene	T53C	F130L	5	3.85%	3	6.12%	8	4.47%	0.511
nt2881–835	T150AC	L162PQ	121	93.08%	10	20.41%	131	73.18%	< 0.001
	C129T	S155L	19	14.62%	0	0	19	10.61%	0.005
	G774A	S207N	43	33.08%	0	0	43	24.02%	< 0.001
	C3026T	A49V	8	6.15%	5	10.20%	13	7.26%	0.352
	T3098C	I73T	3	2.31%	0	0	3	1.68%	0.284
	C3121A	P81T	8	6.15%	0	0	8	4.47%	0.076
	C3189A	D103E	4	3.08%	0	0	4	2.23%	0.214
P gene	A2375C	E23D	6	4.62%	41	83.67%	47	26.26%	< 0.001
nt2307–1623	A2502T	T66S	3	2.31%	17	34.69%	20	11.17%	< 0.001
	C2523G	E73Q	2	1.54%	32	65.31%	34	18.99%	< 0.001
	A37G	S316G	113	86.92%	4	8.16%	117	65.36%	< 0.001
	G49A	V320I	10	7.69%	45	91.84%	55	30.73%	< 0.001
	G886A	V599I	104	80%	0	0	104	58.1%	< 0.001
	G899A	W603Y	0	0	49	100%	49	27.37%	< 0.001
	C930A	Q617DH	4	3.08%	49 ^a^	100%			–
	T1249G	V720L	2	1.54%	46 ^b^	93.88%%			–
	T1351G	S754A	0	0	47	95.92%	47	26.26%	< 0.001
	G1484A	C798Y	0	0	48	97.96%	48	26.82%	< 0.001
	C2712AT	H136YN	97	74.62%	5	10.20%	102	56.98%	< 0.001
	C3051T	P249S	120	92.31%	8	16.33%	128	71.51%	< 0.001
	A3057G	T251A	56	43.08%	3	6.12%	59	32.96%	< 0.001
	A3108G	S268G	43	33.08%	2	4.08%	45	25.14%	< 0.001
	A942T	L613QH	129	99.23%	48 ^c^	97.96%%			–
	T3210A	S272TN	130	100%	49	100%	179	100%	–
X gene	G1386AC	V5ML	31	23.85%	0	0	31	17.32%	< 0.001
nt1374–1838	G1515A, A1516C	D48AN	20	15.38%	0	0	20	11.17%	0.04
	G1613A		14	10.77%	2	4.08%	16	8.94%	0.162
	C1653T	H94Y	7	5.38%	3	6.12%	10	5.59%	0.848
	T1633A	Q87L	2	99.23%	46	93.88%	48	26.82%	< 0.001
	T1485C	S38P	129	0.77%	49	100%	178	99.44%	–

a Amino acid mutation E617DH for CD2

b Amino acid mutation L720VS for CD2

c Amino acid mutation H613K for CD2

* means stop condon

— means *P* value could not be calculated

Compare to reference sequences of genotype D and genotype C, several nucleotide (amino acid) positions changed in nearly all the HBV/CD1 and HBV/CD2 sequences, such as A942T(aaL613QH for HBV/CD1 and aaH613K for HBV/CD2), T1485A and T3210A(aaS272TN) in P gene, T1485C(aaS38P) in X gene.

### Amino acid substitution in PreS/S region

**Fig. 4:**
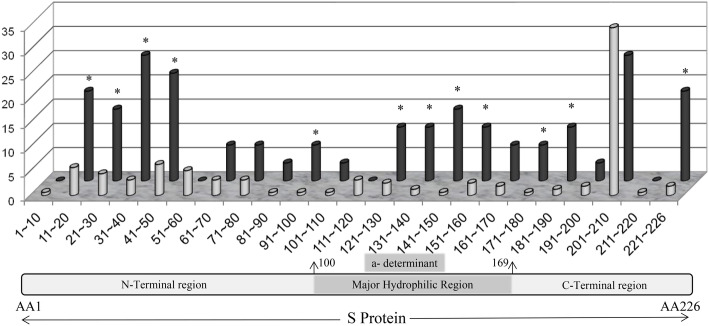
Frequencies of residue substitutions within the S protein. Isolates from HBs Ag/anti-HBs patients (Group I, black bars, *n* = 27) and solely HBsAg-positive patients (Group II, gray bars, *n* = 152) were analyzed in intervals of 10 amino acids each. Each bar represents the percentage of patients with mutated amino acid residues in each group at each interval of 10 amino acids per group. Positions where the proportion of sequences harboring mutations was significant between two groups are marked with an asterisk (*, *P* < 0.05)

One hundred and seventy-nine HBV CD recombinants with complete genome sequences were under analyses of amino acid substitution in PreS/S region. Amino acid substitution of 27 HBsAg+/HBsAb+ strains (Group I) were compared with 152 HBsAg+/HbsAb- strains (Group II). The distribution of different recombination type (HBV/CD1 and HBV/CD2, *P* = 0.677) and HBeAg status (*P* = 0.213) had no significant difference between Group I and Group II. DNA level of the 179 serum samples were all above 5 log10 and had no significant difference between Group I and Group II (*P* = 0.355). Significant aa substitution diversity was observed within S gene of HBV between Group I and Group II (1.03 vs. 0.39, for substitution per 100 aa, the same below, *P* < 0.001). Moreover, the aa variabilities in MHR (*P* < 0.001), “a” determinant (*P* < 0.001), the first loop (*P* < 0.001) and the second loop (*P* < 0.001) of “a” determinant were all more variable in Group I than in Group II. The frequency of PreS deletion was 2.23% (4/179) and also with significant differences between the two groups. Details were listed in Table 4 and Fig. 4.

**Table 4:** Incidence of amino acid mutation/ deletion between HBsAg+/HBsAb+ group (Group I) and HBsAg+/HBsAb- group (Group II)

Section of PreS & HBsAg		Number of substitutions per 100 amino acid residues			
		Group I	Group II	χ^2^	*P* value
PreS1(aa1–118)		0.44	0.26	1.836	0.18
PreS2(aa1–54)		1.92	2	0.190	0.89
PreS deletion^a^		11.11	0.64	11.801	< 0.001
S protein	Full-length of S protein (aa1–226)	1.03	0.31	21.19	< 0.001
	N-terminal (aa1–99)	1.08	0.21	25.6	< 0.001
	MHR (aa100–169)	0.85	0.17	17.6	< 0.001
	a determinant (aa124 ~ 147)	1.54	0.06	30.1	< 0.001
	First loop (aa124–137)	1.59	0.10	20.954	< 0.001
	Second loop (aa139–147)	1.06	0.00	20.804	< 0.001
	C-terminal (aa170 ~ 226)	1.54	0.06	30.1	< 0.001

^a^PreS deletion incidence was calculated by number of deletions per 100 samples

## Discussion

HBV genotypes are related to the severity of liver disease and response to clinical therapy [18]. Compare to other genotypes, HBV genotype C and genotype D carry a higher lifetime risk of liver cirrhosis and hepatocellular carcinoma development [19]. It is believed that recombination can exert an influence on clinical important properties more dramatically than the steady accumulation of natural mutations, which suggests the potential pathogen significance of the HBV/CD recombinants [20]. As far as we know, there was no detailed molecular epidemiology or genetic variability study carried out based on a large number of HBV/CD recombinant complete genome sequences.

In this study, HBV/CD recombinant was the main genotype (179/183, 97.81%) isolated in the plateau. This result is different from recent but smaller sample size reports in Tibet that genotype C and genotype D were the most dominant HBV genotypes [21, 22]. The HBV genotypes showed a distinct geographical distribution all over the world [19]. In both HBV CD1 and CD2 recombinant genomes, the ‘C fragment’ and ‘D fragment’ were genetically close to subgenotype C2 and D4, respectively. HBV/D4 isolates were mainly reported in South America and the Pacific islands [14]. The only report of HBV/D4 in Asia was in northeast India [23]. Specifically, subtype C2 was the main genotype in central and north China [24], so Qinghai-Tibet Plateau was found at the presumed geographical junction to the distribution of these two HBV subgenotypes (Fig. 2). In this study, recombinants CD1 and CD2 had significant different geographic distribution in the plateau. It could also partly explain the different distribution of seromarkers in Tibet and Qinghai. HBV/CD2 isolates were mainly identified in two regions (Shannan & Rikaze) which are close to the boundary of northeast India (Fig. 2). Genotype D fragment was in around nt10–800 of HBV/CD1 and around nt10–1500 of HBV/CD2 (Fig. 1). Then both CD1 and CD2 have the same initial recombination site at nt10. Based on the facts mentioned above, it can be inferred that these two types of recombinations might have a mixed China (subgenotype C2) and Indian (subgenotype D4) origin.

Though lots of HBV genotype D or genotype C isolates were reported in India [23, 25–27], no HBV/CD recombinant strain was reported in south Asia until now. Actually, nearly all the HBV/CD recombinants in this study and in previous studies scattered in indigenous of high altitude areas, such as Qinghai-Tibet Plateau, Yunnan-Kweichow Plateau [28], Loess Plateau [29] and Mongolia Plateau (Accession Number: AB270534, AB270534). A possible reason for this phenomenon might be due to the genetic background or migration history of highlanders.

As the diagnosis information in these rural areas is still not detailed enough to analyze the clinical feature of these HBV recombinants, the complete genome features in this study offer us a way to estimate the clinical prognosis of these HBV strains in the indigenous population. Serotype awy2 was the predominant serotype of HBV/CD in this study, as same as the results of genotype D [30]. It indicates that the main serological character had not dramatically changed after the recombination. Previous studies reported that A1762T/G1764A double mutation in BCP (nt.1742–1849) was the strongest viral factor associated with the development of liver disease [20, 31]. Other mutations, such as T53C, G1613A, C1653T, T1753C, A2189C, T3098C and PreS deletions were also reported associated with clinical progress [32–38]. In this study, the A1762T/G1764A double mutations were observed in 27.93% in HBV CD recombinant sequences. These frequencies were less than previous reports of genotype C in CHB carriers [39], which indicates a lower risk of HCC in the population. The same result was also found in frequencies of mutation A2189C (12.85%), G1613A (8.94%), T1753C (8.38%), C1653T (5.59%), T53C (4.47%), T3098C (1.68%) and PreS deletion (2.23%, 4/179), which were also lower than mutation frequencies of genotype C in CHB patients in Asia [32, 34–40]. This indicates the clinical progress of CD recombinant seems genetically more temperate than genotype C which had caused the most liver disease and related death in China.

There were several mutations frequently identified in PreC/C and PreS/S regions, which potentially influence the clinical prognosis. However, many of these prevalent nucleotide mutations or amino acid substitutions were unique in the CD recombinants, which were not previously reported in genotype C or other genotypes of HBV genome. The function and influence of these mutations need further analysis.

The prevalence of anti-HBs coexistence was 2.14% in the population of HBsAg-positive patients. The coexistence frequency is lower than previous reports [5–7, 41], suggesting possible geographical variability or special recombinant characteristics in these statistical differences. HBeAg status and DNA level of the serum were the focus of the argument about comparability of the HBsAg/HBsAb coexistence studies [8]. In this study, no differences were identified in the distribution of DNA level, HBeAg positivity or recombination types between Group I and Group II. And the DNA level of serum samples in Group I was all above 5 log10 copies/mL. The aa variability of S region in Group I was significantly higher than that in Group II (Table 3, *P* < 0.001), which was consistent with previous reports [41]. In further analysis of each segment in the S region, the aa variability in Group I was significantly increased in N-terminal, MHR and C-terminal, compared with Group II (*P* < 0.001, *P* < 0.001, *P* = 0.013, Fig. 3).

The “a” determinant in MHR is the hot zone of HBsAg/HBsAb coexistence studies, which is located in the hydrophilic region between aa124 and aa147 and act as the most important antigen- binding site in the S region of each HBV serotypes [17, 41]. In this study, compared with Group II, the aa variability in the “a” determinant of Group I increased significantly (1.54% vs 0.06%, *P* < 0.001). This region is composed of two stem- loop structures, which act as two binding sites of monoclonal antibodies and are of great significance for the effectiveness of hepatitis B vaccine and the clinical detection of antigens. In this study, the aa variation of the first and second loop of the “a” determinant were both statistically correlated with HBsAg/HBsAb coexistence, which consistent with previous studies [5, 17]. There were different opinions about the effect of the second loop [42] or even the entire S protein variablity [7] on the HBsAg/HBsAb coexistence. However, former studies were about genotype B and genotype C [42], while HBV/CD recombinant was the main genotype in this study and the “a” determinant was located in genotype D fragment (Fig. 1). It may be due to the amino acid variation background in different genotypes. Moreover, due to the low incidence of HBsAg/ HBsAb coexistence in all HBV genotypes, the sample sizes in previous studies were less than 20 [5, 7], and the insufficient number of samples may affect the stability of statistical results.

The association between aa variation of S protein and HBsAg/HBsAb coexistence has been reported in many studies [5, 6, 17, 41, 42]. However, the aa mutations in any part of S protein is not a sufficient and necessary condition for the explanation of HBsAg/HBsAb coexistence enigma [8], so there should be at least one auxiliary or secondary condition in the emergence of the coexistence. Previous studies suggested that mutations or deletions in the PreS region were the reason for the coexistence of HBsAg/ HBsAb [43]. The significant difference was also found in the distribution of deletions in PreS region between Group I and Group II (Table 3), suggesting the change of PreS region is also associated with coexistence. Interestingly, the frequency of PreS deletion in HBV/CD recombinants was 2.23%, which is lower than the frequency in other genotypes (4.9%) [7]. Combine with the fact that the frequency of HBsAg/HBsAb coexistence is 2.14% in this study, which is also lower than other genotypes [5–7, 41]. PreS deletion may act as a genetic variation background which occasionally affects the interaction between antigens and antibodies, together with the aa mutation in MHR to cause the coexistence.

This study has several limitations. First of all, multiple infections, minor populations of immune escape variants in viral quasispecies may not be identified in this study by PCR product sequencing. Secondly, the combined action of PreS deletion and aa variation in MHR need further support. Finally, the result would be more convincing if we could have a larger sample size of HBsAg/HBsAb coexistence, though 27 HBsAg/HBsAb coexistence in this study is more than most of the previous studies.

## Conclusions

In summary, this study describes the geographical distribution, genetical variability and HBsAg/HBsAb coexistence phenomena of HBV isolates in Qinghai-Tibet plateau. HBV/CD recombinant has become the predominant genotype in Qinghai-Tibet Plateau. There were signs that HBV/CD had a mixed China and South Asia origin. PreS deletion and aa variation in S protein may cause the HBsAg/HBsAb coexistence together. Several unique nucleotide mutations were frequently detected in HBV/CD isolates, which could potentially influence the clinical prognosis.

## Supplementary information

**Additional file 1: Supplementary Table 1.** This table shows the serum markers of 1263 HBV isolates in this study.

**Additional file 2: Supplementary Table 2.** This table shows estimates of Evolutionary Divergence (%) over Sequence Pairs between CD recombinants and subgenotypes D1-D11(nt10–800).

**Additional file 3: Supplementary Table 3.** This table shows estimates of Evolutionary Divergence (%) over Sequence Pairs between CD recombinants and subgenotypes C1-C7(nt1500–9).

**Additional file 4: Supplementary Table 4.** This table shows distribution of HBV subgenotypes in different regions of Qinghai-Tibet Plateau.

## Data Availability

The datasets generated in the current study are based on complete genome sequences which are uploaded by the author in the National Center for Biotechnology Information GenBank database with accession numbers MN683570- MN683729, MN657315- MN657318, KX660674- KX660690.

## Abbreviations

HBV: Hepatitis B Virus
HBsAg: Hepatitis B surface antigen
anti-HBs: Antibody to HBsAg
CHB: Chronic HBV
nt: Nucleotides
HBcAb: Anti-hepatitis B core antibody
HBeAg: Hepatitis B e antigen
HBeAb: Anti-hepatitis B e antibody
PCR: Polymerase chain reaction
BCP: Basal core promoter
MHR: Major hydrophilic region
aa: Amino acid
LC: Liver cirrhosis
HCC: Hepatocellular carcinoma
