# Supplementary material for: Complete genome analysis of hepatitis B virus in Qinghai-Tibet plateau: the geographical distribution, genetic diversity, and co-existence of HBsAg and anti-HBs antibodies
Source: Virol J. 2020 Jun 12;17:75. doi: 10.1186/s12985-020-01350-w (PMC7291583; doi:10.1186/s12985-020-01350-w)
Supplement: Supplementary file 1 — Additional file 1: Supplementary Table 1. This table shows the serum markers of 1263 HBV isolates in this study. [file 12985_2020_1350_MOESM1_ESM.doc]

Supplementary Table 1. Distribution of five serological markers of 1263 HBV isolates in this study

| Location/  Sero-marker | HBsAg | HBsAb | | HBcAb | | HBeAg | | HBeAb | |
| --- | --- | --- | --- | --- | --- | --- | --- | --- | --- |
|  | + | + | - | + | - | + | - | + | - |
| Tibet | 852 | 24 | 828 | 766 | 86 | 331 | 521 | 284 | 568 |
| Qinghai | 411 | 3 | 408 | 411 | 0 | 199 | 212 | 212 | 199 |
| Total | 1263 | 27 | 1236 | 1177 | 86 | 530 | 733 | 496 | 767 |
| *P* value | - | 0.016 | | 0.000 | | 0.001 | | 0.000 | |
