# Supplementary material for: Complete genome analysis of hepatitis B virus in Qinghai-Tibet plateau: the geographical distribution, genetic diversity, and co-existence of HBsAg and anti-HBs antibodies
Source: Virol J. 2020 Jun 12;17:75. doi: 10.1186/s12985-020-01350-w (PMC7291583; doi:10.1186/s12985-020-01350-w)
Supplement: Supplementary file 2 — Additional file 2: Supplementary Table 2. This table shows estimates of Evolutionary Divergence (%) over Sequence Pairs between CD recombinants and subgenotypes D1-D11(nt10–800). [file 12985_2020_1350_MOESM2_ESM.doc]

Supplementary Table 2. Estimates of Evolutionary Divergence (%) over Sequence Pairs between CD recombinants and subgenotypes D1-D11(nt10-800)

| Genotype | D1 | D2 | D3 | D4 | D5 | D6 | D7 | D8 | D9 | D10 | D11 | CD1 | CD2 |
| --- | --- | --- | --- | --- | --- | --- | --- | --- | --- | --- | --- | --- | --- |
| D1 |  |  |  |  |  |  |  |  |  |  |  |  |  |
| D2 | 1.48±0.3 |  |  |  |  |  |  |  |  |  |  |  |  |
| D3 | 1.2±0.3 | 1.49±0.3 |  |  |  |  |  |  |  |  |  |  |  |
| D4 | 1.35±0.3 | 1.77±0.4 | 1.51±0.4 |  |  |  |  |  |  |  |  |  |  |
| D5 | 1.34±0.3 | 1.49±0.3 | 1.26±0.3 | 1.59±0.4 |  |  |  |  |  |  |  |  |  |
| D6 | 1.19±0.3 | 1.65±0.4 | 1.37±0.3 | 0.92±0.2 | 1.46±0.4 |  |  |  |  |  |  |  |  |
| D7 | 1.32±0.3 | 1.69±0.3 | 1.44±0.3 | 0.98±0.2 | 1.5±0.3 | 0.89±0.2 |  |  |  |  |  |  |  |
| D8 | 1.86±0.3 | 2.37±0.4 | 2.09±0.4 | 1.71±0.3 | 2.12±0.4 | 1.33±0.3 | 1.59±0.3 |  |  |  |  |  |  |
| D9 | 1.37±0.3 | 1.62±0.3 | 1.08±0.3 | 1.74±0.4 | 1.16±0.3 | 1.49±0.4 | 1.65±0.4 | 2.19±0.4 |  |  |  |  |  |
| D10 | 1.3±0.2 | 1.56±0.3 | 1.44±0.3 | 1.26±0.3 | 1.53±0.4 | 1.13±0.3 | 1.21±0.2 | 1.89±0.3 | 1.61±0.4 |  |  |  |  |
| D11 | 1.66±0.2 | 2.1±0.3 | 1.83±0.3 | 2.04±0.4 | 2±0.4 | 1.82±0.3 | 1.95±0.3 | 2.53±0.4 | 1.99±0.3 | 1.89±0.3 |  |  |  |
| CD1 | 1.34±0.3 | 1.78±0.4 | 1.5±0.4 | **1.06±0.3** | 1.65±0.4 | 1.46±0.3 | 1.57±0.3 | 2.19±0.4 | 1.75±0.4 | 1.67±0.3 | 1.95±0.3 |  |  |
| CD2 | 1.07±0.3 | 1.5±0.4 | 1.22±0.3 | **0.78±0.3** | 1.39±0.4 | 1.18±0.3 | 1.29±0.3 | 1.92±0.4 | 1.49±0.4 | 1.39±0.3 | 1.66±0.3 | **0.43±0.1** |  |

(Note: The reference genotype strains used in the table are similar to those used in Fig 1. C/D1 and C/D2 were compared with all reference strains; in order to save space, only D1-D11 were shown in this table.)
