# Supplementary material for: Complete genome analysis of hepatitis B virus in Qinghai-Tibet plateau: the geographical distribution, genetic diversity, and co-existence of HBsAg and anti-HBs antibodies
Source: Virol J. 2020 Jun 12;17:75. doi: 10.1186/s12985-020-01350-w (PMC7291583; doi:10.1186/s12985-020-01350-w)
Supplement: Supplementary file 3 — Additional file 3: Supplementary Table 3. This table shows estimates of Evolutionary Divergence (%) over Sequence Pairs between CD recombinants and subgenotypes C1-C7(nt1500–9). [file 12985_2020_1350_MOESM3_ESM.doc]

Supplementary Table 3. Estimates of Evolutionary Divergence (%) over Sequence Pairs between CD recombinants and subgenotypes C1-C7(nt1500-9)

| Genotype | C1 | C2 | C3 | C4 | C5 | C6 | C7 | CD1 | CD2 |
| --- | --- | --- | --- | --- | --- | --- | --- | --- | --- |
| C1 |  |  |  |  |  |  |  |  |  |
| C2 | 2.96 ± 0.3 |  |  |  |  |  |  |  |  |
| C3 | 2.77 ± 0.2 | 2.85 ± 0.3 |  |  |  |  |  |  |  |
| C4 | 4.62 ± 0.4 | 3.35 ± 0.3 | 4.17 ± 0.3 |  |  |  |  |  |  |
| C5 | 5.57 ± 0.5 | 4.67 ± 0.5 | 5.42 ± 0.5 | 6.09 ± 0.5 |  |  |  |  |  |
| C6 | 4.55 ± 0.4 | 3.53 ± 0.4 | 4.05 ± 0.4 | 4.08 ± 0.3 | 5.97 ± 0.6 |  |  |  |  |
| C7 | 4.3 ± 0.3 | 3.36 ± 0.3 | 4.04 ± 0.3 | 4.5 ± 0.3 | 4.44 ± 0.4 | 4.32 ± 0.4 |  |  |  |
| CD1 | 2.77 ± 0.3 | **1.19 ± 0.1** | 2.61 ± 0.3 | 3.21 ± 0.3 | 4.53 ± 0.5 | 3.37 ± 0.4 | 3.16 ± 0.3 |  |  |
| CD2 | 3.15 ± 0.3 | **1.5 ± 0.2** | 2.97 ± 0.3 | 3.39 ± 0.3 | 4.79 ± 0.5 | 3.49 ± 0.4 | 3.49 ± 0.3 | **1.07 ± 0.2** |  |

(Note: The reference genotype strains used in the table are similar to those used in Fig 1. C/D1 and C/D2 were compared with all reference strains; in order to save space, only C1-C7 were shown in this table.)
