# Supplementary material for: Complete genome analysis of hepatitis B virus in Qinghai-Tibet plateau: the geographical distribution, genetic diversity, and co-existence of HBsAg and anti-HBs antibodies
Source: Virol J. 2020 Jun 12;17:75. doi: 10.1186/s12985-020-01350-w (PMC7291583; doi:10.1186/s12985-020-01350-w)
Supplement: Supplementary file 4 — Additional file 4: Supplementary Table 4. This table shows distribution of HBV subgenotypes in different regions of Qinghai-Tibet Plateau. [file 12985_2020_1350_MOESM4_ESM.doc]

Supplementary Table 4. Distribution of HBV subgenotype in different regions of Qinghai-Tibet Plateau

| Location/Subgenotype | CD1(130 isolates)  No.& % | | CD1(130 isolates)  No.& % | | C2（4 isolates）  No.&% | | Sum |
| --- | --- | --- | --- | --- | --- | --- | --- |
| Lhasa | 13 | 81.25 | 2 | 12.5 | 1 | 6.25 | 16 |
| Rikaze | 7 | 21.21 | 26 | 78.79 | 0 | 0 | 33 |
| Chamdo | 35 | 97.22 | 1 | 2.78 | 0 | 0 | 36 |
| Nyingchi | 11 | 91.67 | 0 | 0 | 1 | 8.33 | 12 |
| Shannan | 10 | 33.33 | 20 | 66.67 | 0 | 0 | 30 |
| Nakqu | 18 | 100 | 0 | 0 | 0 | 0 | 18 |
| Ali | 20 | 100 | 0 | 0 | 0 | 0 | 20 |
| Hainan | 16 | 88.89 | 0 | 0 | 2 | 11.11 | 18 |
